# Concurrent chemo-radiotherapy following neoadjuvant chemotherapy in locally advanced breast cancer

**DOI:** 10.1186/1748-717X-4-24

**Published:** 2009-07-11

**Authors:** Alberto Alvarado-Miranda, Oscar Arrieta, Carlos Gamboa-Vignolle, David Saavedra-Perez, Rafael Morales-Barrera, Enrique Bargallo-Rocha, Juan Zinser-Sierra, Victor Perez-Sanchez, Teresa Ramirez-Ugalde, Fernando Lara-Medina

**Affiliations:** 1 Department of Medical Oncology, Instituto Nacional de Cancerologia, Mexico City, Mexico; 2 Department of Radiotherapy, Instituto Nacional de Cancerologia, Mexico City, Mexico; 3 Department of Breast Tumors, Instituto Nacional de Cancerologia, Mexico City, Mexico; 4 Department of Pathology, Instituto Nacional de Cancerologia, Mexico City, Mexico

## Abstract

**Background:**

Despite broad advances in multimodal treatment of locally advanced breast cancer (LABC), 30 to 40% of patients develop loco-regional relapse. The aim of this study was to analyze in a retrospective manner the effectiveness of concurrent chemo-radiotherapy (CCRTh) after neoadjuvant chemotherapy (NCT) in patients with LABC.

**Methods:**

One hundred twelve patients with LABC (stage IIB-IIIB) were treated with NCT (5-fluorouracil 500 mg/m^2^, doxorubicin 50 mg/m^2^, and cyclophosphamide 500 mg/m^2 ^(FAC), or doxorubicin 50 mg/m^2 ^and cyclophosphamide 500 mg/m^2 ^(AC) IV in four 21-day courses) followed by CCRTh (60 Gy breast irradiation and weekly mitomycin 5 mg/m^2^, 5-fluorouracil 500 mg/m^2^, and dexamethasone 16 mg, or cisplatin 30 mg/m^2^, gemcitabine 100 mg/m^2 ^and dexamethasone 16 mg), and 6–8 weeks later, surgery and two additional courses of FAC, AC, or paclitaxel 90 mg/m^2 ^weekly for 12 weeks, and in case of estrogen-receptor positive patients, hormonal therapy.

**Results:**

Stages IIB, IIIA and -B were 21.4, 42.9, and 35.7%, respectively. Pathological complete response (pCR) in the breast was 42% (95% CI, 33.2–50.5%) and, 29.5% (95% CI, 21.4–37.5%) if including both the breast and the axillary nodes. Multivariate analysis showed that the main determinant of pCR was negative estrogen-receptor status (HR = 3.8; 95% CI, 1.5–9; *p *= 0.016). The 5-year disease-free survival (DFS) was 76.9% (95% CI, 68.2–84.7%). No relationship between pCR and DFS was found. Multivariate analysis demonstrated that the main DFS determinant was clinical stage (IIB and IIIA *vs. *IIIB, HR = 3.1; 95% CI, 1.02–9.74; *p *= 0.04). Only one patient had local recurrence. Five-year overall survival was 84.2% (95% CI, 75–93.2%). The toxicity profile was acceptable.

**Conclusion:**

This non-conventional multimodal treatment has good loco-regional control for LABC. Randomized clinical trials of preoperative CCRTh following chemotherapy, in patients with LABC are warranted.

## Background

Breast cancer is the second leading cause of cancer death among women in developed countries and in Mexico [1,2]. In Mexico, only a small percentage of women have regular mammography screening; therefore the proportion of patients with locally advanced disease at diagnosis is high. In 2003, only 5–10% of newly diagnosed cases in Mexico were clinical stages 0 or I [2]. In fact, nearly 70% of breast cancer cases of patients seen at the Instituto Nacional de Cancerología (INCan: a cancer-referral teaching hospital for adult patients, located in Mexico City) are stage IIB-IIIB, locally advanced breast cancer (LABC, 6th edition of the AJCC Cancer Staging) [3,4].

Current treatment of LABC requires a combination of systemic chemotherapy (CT), surgery, and radiotherapy (RT) [5]. Between six and eight courses of anthracycline- and taxane-based regimens administered sequentially or in combination are now recommended, and CT should be followed by segmental or modified radical mastectomy for operable tumors. Patients with inoperable tumors after maximal CT (i.e., taxane if initial therapy was anthracycline-based) could proceed to definitive RT. Patients treated with surgery should receive post-operative RT to minimize the risk of local recurrence. In addition, women with hormone-receptor-positive tumors should receive hormonal therapy (HT) [5].

However, only 10–20% of patients with LABC achieve clinical complete response, and 50–60%, partial response [6-8]. Pathological complete response (pCR) rate in LABC is poor, 8–12%, and often does not correlate with clinical response [6,8-11]. Approximately, 30–40% of patients with LABC develop loco-regional relapse (LRR) [8].

Despite improvements in local control rates and overall outcomes with current therapy, 5-year survival for LABC remains low (50% *vs. *87% for stage I) [12]. Moreover, concurrent chemo-radiotherapy (CCRTh) with anthracycline drugs is theoretically more toxic; therefore, in patients with LABC, this treatment modality has not been widely used. However, CCRTh has successfully improved both local control and overall survival (OS) in other cancers such as esophageal, lung, head and neck, and cervix [13-16].

The aim of this study was to determine disease-free survival (DFS), pathologic complete response (pCR) and associated factors using a multimodal therapy (neoadjuvant chemotherapy followed by concurrent CCRTh, surgery, and CT) in patients with locally advanced breast cancer.

## Methods

### Patients and samples

From January 2000 to December 2003, patients seen at the INCan Department of Breast Tumors with diagnosis of breast cancer confirmed by histopathology who presented loco regional disease (stages IIB, IIIA and -B, according to the 6th edition of the American Joint Committee on Cancer TNM classsification and staging system and evaluated by thoracic computed tomography (CT) scans, bone scinthigraphy and/or PET-CT) [4], without clinical response (according to the attending physician, based on an increase in the breast tumor and/or pathologic axillary lymph node diameters ≥50%) after completion of anthracycline-containing neoadjuvant chemotherapy, and without evidence of distant metastases at diagnosis were enrolled; this primary CT was followed by concurrent chemo-radiotherapy (CCRTh), modified radical mastectomy, and adjuvant systemic treatment. Exclusion criteria comprised other clinical stages from IIB, IIIA, and -B, Phyllodes tumour as histological diagnosis, and treatment variations. Biopsies were examined and classified by a pathologist specialized in this tumor type. The Breast Cancer Classification proposed by the World Health Organization was employed to classify each biopsy. We utilized the Scarff-Bloom-Richardson (SBR) scale that is based on nuclear pleomorphism and mitotic count, to stratify each tumor's tissue differentiation. Hormonal status was obtained by immunohistochemistry on sections of formalin-fixed, paraffin-embedded tissue, from incisional biopsies and subsequent surgical specimens.

### Treatment

Neoadjuvant CT was instituted in four 21-days courses. The following two treatment schedules were utilized: a) 5-fluorouracil (500 mg/m^2^), adriamycin (50 mg/m^2^), and cyclophosphamide (500 mg/m^2^) (FAC), or b) adriamycin (50 mg/m^2^) and cyclophosphamide (500 mg/m^2^) (AC). CCRTh after the previously mentioned regimen was as follows: RT 60 Gy (3-D CT-based simulation) to the whole-breast and nodal areas divided into 50 Gy in 5 weeks plus boost to palpable residual disease with a 10 Gy electron beam in 1 week, and CT based on mitomycin C (5 mg/m^2^), 5-fluorouracil (500 mg/m^2^), and dexamethasone (16 mg), or cisplatin (30 mg/m^2^), gemcitabine (100 mg/m^2^) and dexamethasone (16 mg), weekly during RT (six cycles in total). Radiation Therapy Oncology Group (RTOG) scale was used for toxicity assessment.

Modified radical mastectomy and axillary lymph-node dissection were performed post-CCRTh. Six to eight weeks after surgery, patients received adjuvent systemic treatment with FAC or AC for two additional courses, as previously described. Patients in another subgroup were treated with paclitaxel at a dose of 90 mg/m^2 ^weekly for 12 weeks. Adjuvant hormonal treatment was administered to patients with positive tissue hormonal receptors.

### Response

Pathological complete response (pCR) was defined as no presence of tumor or microscopic disease (presence of microscopic foci in histologic sample) in breast (pCRB) samples, resected axillary (pCRA) lymph nodes and both sites (pCR). Pathological response was classified as residual if any tumour was present.

### Statistical analysis

For descriptive purposes, continuous variables were summarized as arithmetic means and standard deviations (SDs, errors), and categorical variables comprised relative frequencies and proportions. Inferential comparisons were performed with the Student *t *test or the Mann-Whitney *U *test according to the distribution (normal and non-normal) determined by the Kolmogorov-Smirnov test. The Chi-squared or Fisher exact test was utilized to compare clinical variables and pCR. Logistical regression analysis was employed in significant (or near significant; *p *= 0.1) variables. Disease-free (DFS) and overall survivals (OS) were analyzed with the Kaplan-Meier technique, and comparisons among subgroups were performed with a log-rank test. All variables were dichotomized for survival analysis. Adjustment of potential confounders was conducted by log-rank analysis stratification and by Cox proportional hazards regression multivariate analysis. All tests were two-sided, and the significance value was set at *p *= 0.05. SPSS (version 10.0; SPSS, Inc., Chicago, IL, USA) and STATA (StataCorp, College Station, TX) software packages were employed for data analysis.

## Results

### Patients and Samples

Between January 2000 and December 2003, 112 patients met the selection criteria for this study. Mean age was 50 ± 11 years, and median tumour size was 5 ± 1.56 cm. Patients with tumor at stages T2, -3 and -4 represented 19.6, 44.6 and 35.7% of cases, respectively. Thus, patients were in clinical stages IIB, IIIA, and -B were 21.4, 42.9, and 35.7%, respectively (Table 1). All neoplasms were infiltrating ductal carcinoma. ER-positive expression was found in 42.9%, and in 41.1% for PgR. Low/moderate histological grade was 40.2, while and high grade stood at 59.8%. Human epithelial growth factor receptor 2 (HER2) expression was not included in data analysis because only two patients (1.7%) were positive. Post-surgical systemic treatment was based on anthracycline in 48.2% and on taxane drugs in 51.8% of patients (Table 1).

**Table 1:** Baseline patient characteristics

**Variable** ***N *= 112**	**Median ± SE**	**Number** **(%)**
**Age **(years)	50 ± 11	
		
**Tumor size** **(cm)**	5 ± 1.56 Mean, 3.93	
		
Clinical T stage*		
T2		22 (19.6)
T3		50 (44.6)
T4		40 (35.7)
		
Clinical N stage*		
N1		55 (49.1)
N2		56 (50)
N3		1 (0.9)
		
Clinical stage*		
IIB		24 (21.4)
IIIA		48 (42.9)
IIIB		40 (35.7)
		
**Histological grade†**		
Low/moderate		45 (40.2)
High		67 (59.8)
		
**Estrogen receptors**		
Positive		48 (42.9)
Negative		64 (57.1)
		
**Progesterone receptors**		
Positive		46 (41.1)
Negative		66 (58.9)
		
**Treatment after CCRTh**		
Anthracycline		54 (48.2)
Taxanes		58 (51.8)

*Abbreviations*: SE = standard error; CCRTh = concurrent chemo-radiotherapy.

* 6th edition of the American Joint Committee on Cancer TNM classification

and staging system.

† Scarff-Bloom-Richardson (SBR) scale.

### Pathological Complete Response

Pathological response was independently assessed in the primary site and in the axillary lymph nodes. In the breast, pCRB was present in 42% (95% Confidence interval [95% CI], 33.2–50.5), microscopic disease in 27.7% (95% CI, 19.2–36.8), and residual disease in 30.4% (95% CI, 25.3–38.9) of patients, while in the axilla, pCRA was found in 58% (95% CI, 52.8–65.1) and persistent disease in 42% (95% CI, 30.2–47.3) of patients. At both sites (breast and axilla), pCR was 29.5% (95% CI, 21.4–37.5) (Table 2). Table 3 shows the relationship between pCR and clinico-pathological factors. Multivariable analysis demonstrated that the main determinant of pCR was negative ER status (HR = 3.8; 95% CI, 1.5–9; *p *= 0.016).

**Table 2:** Frequency of pathological-complete response at primary site and axilla

**Primary site/axilla** ***N *= 112**	**Frequency** **(%)**
Negative/negative	29.5
Negative/positive	12.5
Positive/negative	28.6
Positive/positive	29.5

**Table 3:** Relationship between pathological-complete response at breast and axilla with clinico-pathological factors

**Variable** *N *= 112	**pCR** **% (95% CI)**	**Univariate analysis** ***p***	**Multivariate analysis** **HR (95% CI)** ***p***
**Age **(years)		0.204	
>50	64 (54–74)		
<50	75 (67–83)		
			
Clinical T stage*		0.218	
T2	54 (45–62)		
T3	72 (64–80)		
T4	75 (67–83)		
			
Clinical N stage*		0.072	
N1	59(51–67)		
N2/N3	78 (70–85)		
			
Clinical stage*		0.656	
IIB	66 (58–74)		
IIIA	66 (58–74)		
IIIB	75 (67–83)		
			
**Estrogen receptors**		0.002	3.8 (0.149–0.087) 0.016
Negative	81 (74–88)		
Positive	54 (45–63)		
			
**Progesterone receptors**		0.090	1.1 (0.391–3.571) 0.767
Negative	75 (67–83)		
Positive	60 (52–68)		
			
**Histological grade†**		0.06	0.5 (0.244–1.038,) 0.063
Low/moderate	60 (52–68)		
High	76 (68–84)		

*Abbreviations*: pCR = pathological complete response; 95% CI = 95% confidence interval; HR = hazard ratio.

* 6th edition of the American Joint Committee on Cancer TNM classification and staging system.

† Scarff-Bloom-Richardson (SBR) scale.

### Outcomes

Mean follow-up was 43 months (range 7–125 months). Median DSF has not been achieved. Five-year DFS was 76.9% (95% CI, 68.2–84.7%). No relationship between pCR and DFS was found. As independent factors, clinical stages IIB and IIIA were associated with a longer disease-free survival (HR = 3.1; 95% CI, 1.02–9.74; *p *= 0.04) as compared to clinical stage IIIB (Table 4). Only one patient had local recurrence. Tumor relapse occurred in 12.5, 3.6, 2.7, and 1.8% as bone, lung, liver and brain metastasis, respectively. Three patients had more than one recurrence site. OS at 5 years was 84.2% (95% CI, 75–93.2%). Toxicity exhibited during CCRTh was as follows: grade 1–2 neutropenia in 32.2%, grade 1–2 anemia in 5.2%, and grade 3 radioepithelitis in 22.4% of patients.

**Table 4:** Relationship between disease-free survival with clinico-pathological factors

**Variable** ***N *= 112**	**1-year DFS** (months ± SD)	**2-year DFS** (months ± SD)	**5-year DFS** (months ± SD)	**Univariate analysis** ***p***	**Multivariate analysis** **HR (95% CI)** ***p***
**Age **(years)				0.09	
>50	92 ± 3	82 ± 5	82 ± 5		
<50	96 ± 2	94 ± 2	92 ± 3		
					
Clinical T stage*				0.05	
T2	90 ± 6	86 ± 7	86 ± 7		
T3	98 ± 2	96 ± 2	96 ± 2		
T4	87 ± 5	82 ± 6	78 ± 6		
					
Clinical N stage*				0.79	
N1	92 ± 3	90 ± 4	90 ± 4		
N2/N3	92 ± 3	87 ± 4	84 ± 5		
					
Clinical stage*				0.03	3.1 (1.02–9.74,) 0.0406
IIB/IIIA	95 ± 2	93 ± 3	93 ± 3		
IIIB	87 ± 5	82 ± 6	78 ± 6		
					
**Estrogen receptors**				0.012	0.3 (0.91–1.22,) 0.97
Negative	92 ± 3	85 ± 4	83 ± 4		
Positive	97 ± 2	93 ± 3	93 ± 3		
					
**Histological grade†**				0.04	3.5 (0.79–16.28,) 0.98
Low/moderate	100	97 ± 2	94 ± 3		
High	91 ± 3	83 ± 4	83 ± 4		
					
**Pathological response**				0.56	
pCR/microscopic	92 ± 3	88 ± 3	86 ± 4		
Residual	94 ± 4	91 ± 4	91 ± 4		

*Abbreviations*: DFS = disease-free survival; SD = standard deviation; HR = Hazard ratio; 95% CI = 95% confidence interval.

* 6th edition of the American Joint Committee on Cancer TNM classification and staging system.

† Scarff-Bloom-Richardson (SBR) scale.

## Discussion

Use of neoadjuvant systemic CT and post-mastectomy RT has become standard for patients with LABC because this treatment course improves prognosis substantially and enhances the possibility of surgery [7,17,18]. Advances in neoadjuvant systemic CT for LABC include not only earlier treatment of sub-clinical distant micrometastases and primary-tumour downstaging, but also the possibility of *in vivo *assessment of response to specific systemic agents. Thus, it is not only rational, but also current practice, to apply this approach in inoperable LABC [19]. However, the magnitude of benefit from neoadjuvant CT on survival in breast cancer remains unclear due to the few comparative trials conducted specifically on LABC [19]. Comparative trials of neoadjuvant *vs. *adjuvant CT in primary operable breast cancer demonstrate equivalent survival outcomes [7]. Despite multimodal therapy improvements in LABC, 11–30% of patients develop local relapse [5,20]. Moreover, poor reponse to neoadjuvant CT is known to be associated with a higher probability of loco-regional recurrence (LRR) [20]. In our study, no patient responded to neoadjuvant CT; thus, patients presented a high risk for LRR. Additionally, approximately 60% were ER-negative, which represents an additional risk factor for patients with LRR in LABC treated with neoadjuvant chemotherapy, mastectomy, and RT [20].

Clinical trials regarding the role and benefit of RT in the management of patients with LABC are sparse. The limited data and guidelines available do suggest that loco-regional RT should be employed in post-mastectomy LABC to reduce LRR rates [17,18,21,22]. We administered first neoadjuvant chemotherapy, because it is the standard treatment for LABC. Nevertheless, all included patients did not present clinical response, thus we proposed CCRTh. We employed two regimens of CCRTh. The first was based on 5-FU and mitomycin C, because of previously good reported results with this treatment in patients with anal carcinoma [23]. The second regimen was based on cisplatin and gemcitabine, because of good results with this multimodal treatment in head and neck carcinoma and cervical cancer reported in our Institution [24,25]. Moreover, we added dexamethasone to these two regimens as an antiemetic drug, and to reduce the risk of radiation neumonitis. Despite the recent knowledge of the higher radiation-neumonitis frequency in patients with lung cancer treated with radiotherapy combined with gemcitabine [26,27], none of enrolled patients developed severe lung or cardiac toxicity as late effects. Moreover, a phase I study showed a reduction of local recurrence rate with the addition of gemcitabine to chemotherapy in unresectable chest wall recurrences [28].

Many issues remain unclear, such as best timing for radiotherapy in relation to surgery. We added CCRTh to standard anthracycline-based chemotherapy to improve local control in this group of patients, obtaining a 5-years DFS of 76.9% and only one LRR among these 112 patients (1%). RT as pre-operative or unique modality has been described for some time with variable outcomes and reports of 5-year clinical cure in different breast-cancer clinical stages [29-31]. In another retrospective study, pre-operative RT was administered to 75 patients with tumors >3 cm and only 12% developed LRR, nearly all patients (96%) underwent conservative surgery with satisfactory cosmetic results [30]. In contrast, in the present study the all patients underwent mastectomy, which likely contributed to the lower LRR (1%) observed in this study.

There are few reports of CCRTh in LABC. Additional experience in treatment type is available for early stage breast carcinoma [31-33]. A retrospective study analyzed 38 patients from five institutional trials with inoperable loco-regional disease after primary chemotherapy completion and pre-operative RT treatment, reporting a 5-years DFS of only 35% and a 5-year LRR of 27% for surgically treated patients. In our study we report longer survival and progression-free rates among a larger cohort of patients. Differences between our results and those of the previously mentioned study could be due to differences in the patient populations (our study did not include stage IV patients, while 24% of patient in the other study had N3 disease) and our use of CCRTh with radiosensitizing agents (mitomycin C (5 mg/m^2^), 5-fluorouracil (500 mg/m^2^), and dexamethasone (16 mg) or cisplatin (30 mg/m^2^), gemcitabine (100 mg/m^2^), and dexamethasone (16 mg) weekly for six total courses) during RT.

The success of RT depends on increasing malignant-cell sensitivity to radiation-induced cell kill coupled with a reduction in metastasis phenotypes of these cells. Radiation damage to cells and tissues involve generation of reactive oxygen species and reactive nitrogen species followed by alterations in lipids, DNA, and proteins, which eventually lead to cellular dysfunction or cell death. Alterations in lipid membrane due to peroxidative damage may form a potential initiator of radiosensitizing effects in combination with drugs acting through modulation of membrane associated events involved in apoptosis induction and increasing oxidative damage or by synchronizing cells to a radiosensitive phase of the cell cycle thus causing enhanced killing [34]. This is the rationale for utilizing radiosensitizing agents, and could explain the good pathological response and LRR rates of our study. Nonetheless, this treatment type could increase toxicity as a result of cell damage and apoptosis, but this event was presented in our study patients with the following acceptable profile: grade 1–2 neutropenia in 32.2%; grade 1–2 anemia in 5.2%, and grade 3 radioepithelitis in 22.4% of patients. This toxicity is consistent with other retrospective analyses on CCRTh, but in patients with early breast carcinoma [32,33]. For example, a retrospective analysis of 106 patients with early disease treated with CCRTh after breast conservative surgery (adjuvant CCRTh) reported grade 3 radioepithelitis in 20% of patients. Furthermore, when authors compared sequential CT and RT with CCRTh, the latter treatment was superior for 10-year local control (92 *vs. *83%); however, in this report there were at least four different CCRTh schedules; therefore, it is difficult to conclude which of the four comprises the better treatment regime [33]. Another retrospective study compared 485 patients treated with conservative surgery and post-operative RT with or without concurrent CT, and reported at multivariate analysis that the CCRTh group exhibited a statistically lower recurrence rate with significantly higher grade 2 acute skin toxicity in the concurrent group (21.2 *vs. *11.2% of the RT-only group; *p *< 0.0001) [32]. A phase III study compared concurrent or sequential adjuvant CRT after conservative surgery for early-stage breast cancer, reporting no significant difference for DFS or LRR-free survival; nevertheless in the node-positive subgroup, the 5-year LRR-free survival was statistically better in the concurrent arm (97% in concurrent *vs. *91% in sequential; *p *= 0.02) corresponding 39% decreased risk for LRR [35].

In our study, on multivariate analysis, ER negative tumors were associated with higher pCR rates, and poorly differentiated tumors showed a trend for higher pCR rates. A study of 399 pre-operative CT-treated patients with LABC reported that negative ER- and PgR expression and grade 3 are associated with high pCR rates [36]. Two other studies reported similar results concerning the association of absent hormonal receptors (12 times more likely to achieve a pCR) and high histological grade with major pCR rate to neoadjuvant CT in patients with LABC [9,37]. Response rates of neoadjuvant CT in LABC are between 5 and 8.7% with anthracycline-based CT, taxane-containing regimens, or navelbine-containing regimens [9,37,38]. In our study, using CCRTh, we found superior pRCB (42%) and pCR (29.5%) than in other series. A phase II study reported similar results to ours, for example, a 27% pCR rate employing pre-operative CCRTh for breast cancer, in which CT was based on 5-FU and vinorelbine regimens. Therefore, similar to our results, these authors found three pCR-associated factors: histological grade 3; absence of hormonal receptors, and high mitotic index [39]. Tumour response to pre-operative CT correlates with outcomes and could identify patients with CT-sensitive micrometastases [7]. We found no association between pCR and DFS. A possible explanation is that tumor response to CCRTh does not reflect sensitivity systemically, but only locally.

A previous report clearly describes the surgical complications of CCRTh-treated patients at our Institution. Three hundred sixty patients were enrolled in this report, of whom 46% developed wound complications, 17% surgical site infection, and 16.9% developed necrosis. The authors found that radiotherapy-induced skin toxicity comprises a risk factor for development of major wound complications. These elevated wound complications may be explained by radiotherapy effects on tissue healing, decrease of vascularity, and induction of tissue-hypoxia and fibrosis, producing necrosis and ulceration [3]. In our analysis, we only included 112 of these 360 patients, because they achieved selection criteria for our analysis. Despite that our series has the larger reported number of CCRTh-treated patients and that treatment was homogeneous (only two regimens of radiosensitizing CT), it entertains the limitation of being a retrospective analysis and patient selection was based on clinical response according to the attending physician. Notwithstanding this, we describe valuable information regarding the CCRTh effect and toxicity in patients with high recurrence risk.

## Conclusion

In summary, our results suggest that CCRTh following neoadjuvant chemotherapy possesses good local control with an acceptable toxicity profile, despite the poorer prognosis of patients with inoperable disease after primary chemotherapy in LABC. However, a prospective study needs to be developed to evaluate chemotherapy effectiveness followed by concomitant chemoradiotherapy as induction in the group of patients with high recurrence risk.

## Competing interests

The authors declare that they have no competing interests.

## Authors' contributions

AAM, RMB, EBR, JSZ, TRU, and FLM participated in the design and follow-up of patients. CGB participated in the design, follow-up and radiological treatment of patients. VPS performed the analysis of tumor specimens. OA and DSP performed the statistical analysis and helped to draft the manuscript. FLM conceived of the study, and participated in its design and coordination and helped to draft the manuscript. All authors read and approved the final manuscript.
